# Supplementary material for: Ultrablack wool textiles inspired by hierarchical avian structure
Source: Nat Commun. 2025 Nov 26;16:10581. doi: 10.1038/s41467-025-65649-4 (PMC12657505; doi:10.1038/s41467-025-65649-4)
Supplement: Supplementary file 1 — Supplementary Information [file 41467_2025_65649_MOESM1_ESM.pdf]

Supplementary Information for

Ultrablack Wool Textiles Inspired by Hierarchical Avian Structure

Hansadi Jayamaha†, Kyuin Park†, Larissa M. Shepherd*

Department of Human Centered Design, Cornell University, Ithaca, New York 14853, United States

† These authors contributed equally

* Correspondence to: larissa.shepherd@cornell.edu

Supplementary Figures

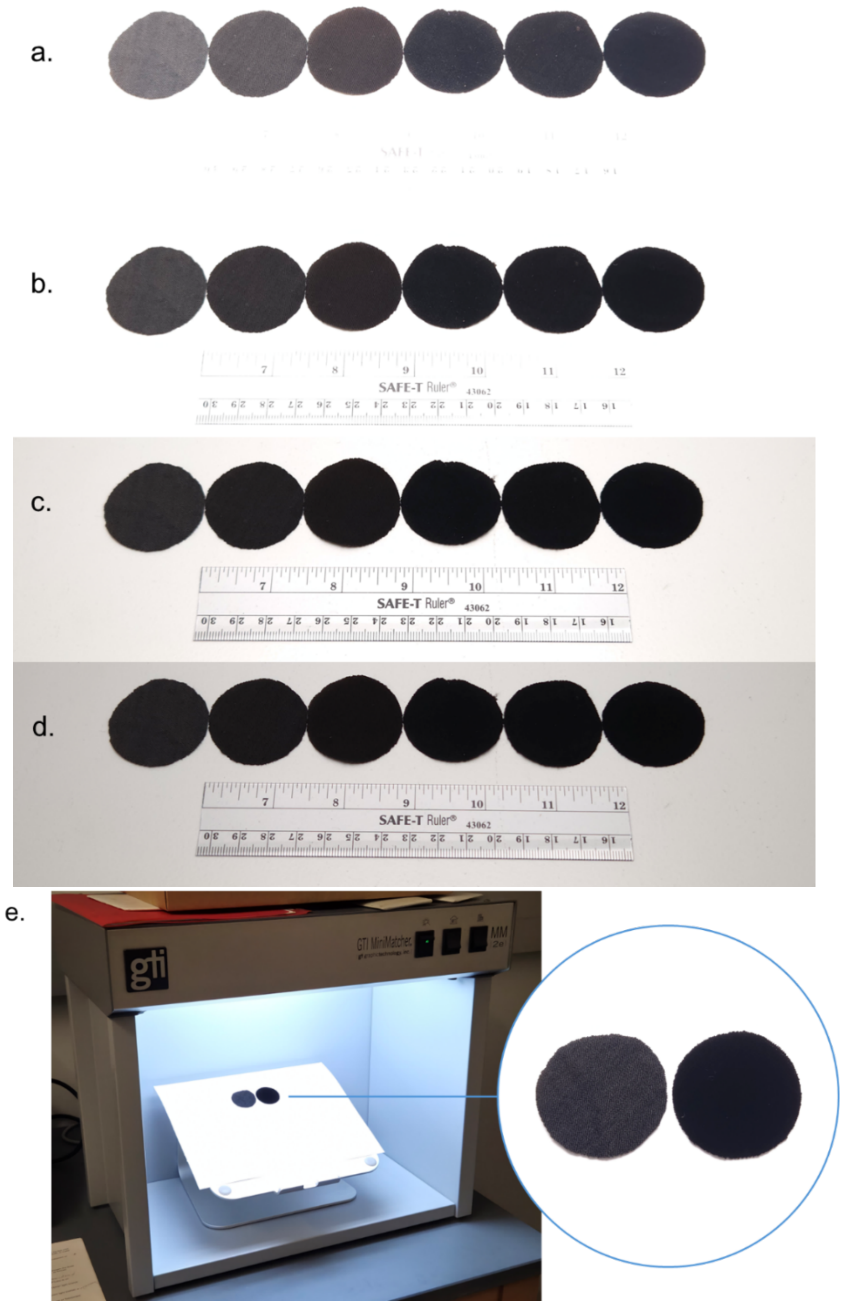

Supplementary Figure 1. Example photographic image of D65 (Daylight) standard lighting. (**a-d**) PDA-dyed merino wool samples after different plasma treatment times under D65 lighting. Plasma treatment times (0, 5, 15, 25, 40, 80 min) are shown from left to right. L* values are 14.54, 9.24, 4.54, 2.87, 0.99, and 0.55, respectively. Camera settings were fixed to F1.8, ISO 50, WB 6000K. Shutter speed: **a.** 1/6 sec, **b.** 1/15 sec, **c.** 1/30 sec, **d.** 1/45 sec. **e.** Fabric samples are PDA dyed merino wool (left) and UBW (right).

GTI Mini Matcher MM-2e meets the quality, intensity, evenness, and geometry of lighting standard of ASTM D1729 “Standard Practice for Visual Appraisal of Colors and Color Differences of Diffusely Illuminated Opaque Materials”.

Photographic images of fabric samples were captured under D65 (daylight) illumination, following the general guidelines of AATCC EP9 "Evaluation Procedure for Visual Assessment of Color Difference of Textiles," with specific modifications to the viewing angle and distance. For example, for Figure 1c, viewing geometry Option A was employed, with illumination incident at a 45° tilt from the base; Supplementary Figure 1 a-d were captured using viewing geometry Option B, which involved illumination incident at 90° (direct illumination) with observation from 45°; and Figures 1e was obtained using a modified viewing geometry based on Option C, where illumination was incident at ~30°, and fabric samples were positioned closer to the illumination source, same as shown above in Supplementary Figure 1e.

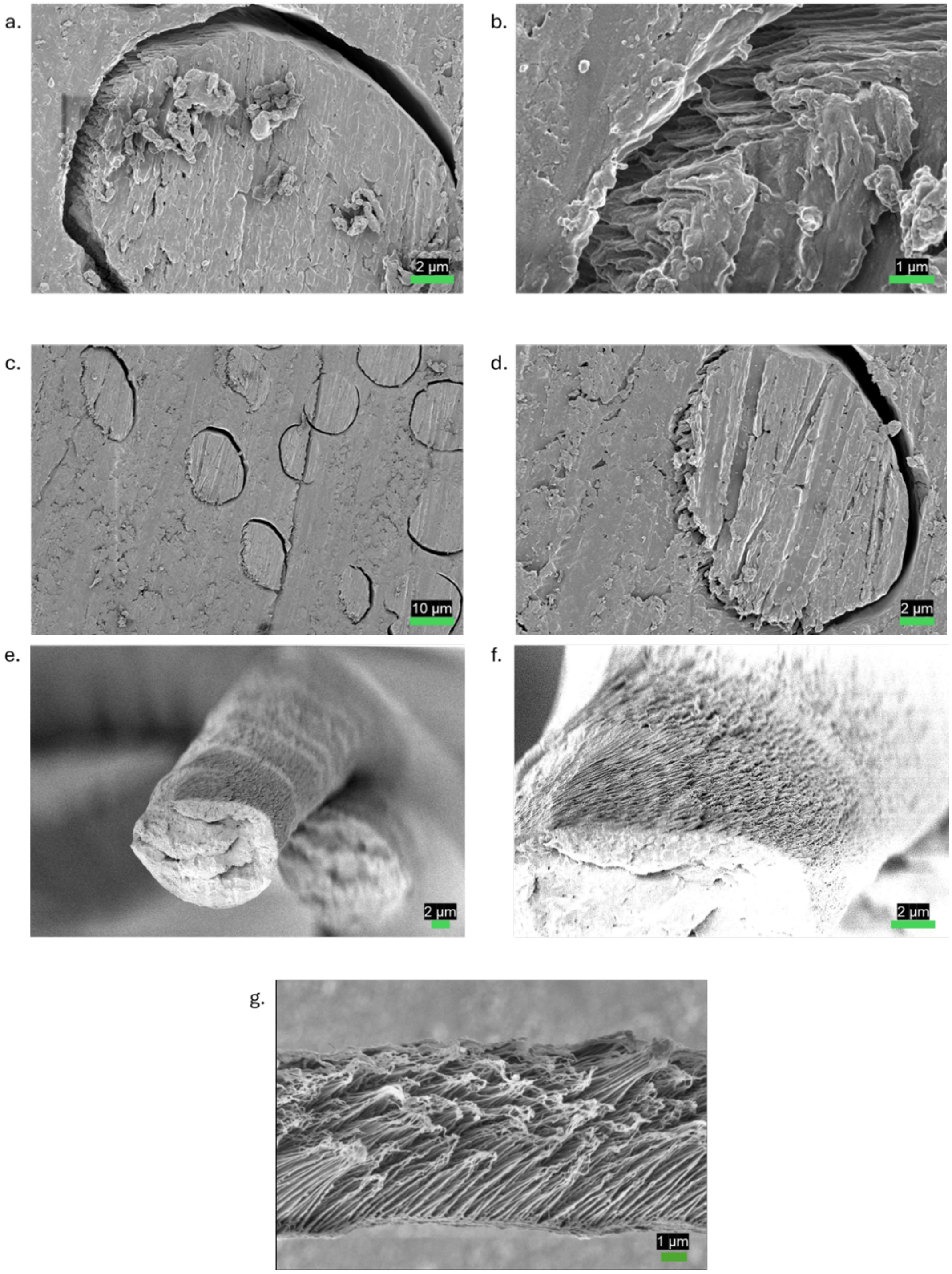

Supplementary Figure 2. **a.** Wool fiber of UBW embedded in epoxy. **b.** Nanopillars of '**a**' exhibiting a directional orientation while following the contour of the fiber. **c.** Nanostructured fibers on the face of UBW contrasting from smooth fibers underneath the outermost layer that was exposed to plasma etching. **d.** Zoomed in image of a nanostructured fiber from **c**. **e, f.** Freeze-fractured UBW fiber. **g.** side-view of UBW fiber. These images confirm that the bundles are formed from nanopillars. Also, the height of the bundles can vary from the edge to the center of the treated surface.

Cross-sectional scanning electron microscopy (SEM) images of ultrablack wool (UBW) fabrics were prepared using two distinct methods: (a-d) embedding in epoxy followed by mechanical polishing, and (e, f) freeze-fracturing with liquid nitrogen. These images illustrate three key phenomena resulting from plasma treatment: (1) Plasma etching is confined to the exposed surface on the face of the fabric. (2) Nanostructures/nanopillars exhibit directional alignment with the plasma, rather than forming with random orientation. (3) Nanopillars on the fiber surface follow the contour of the fiber, providing 3-dimensional surface for light-trapping when compared to nanostructures on a flat surface. These SEM images, however, do not clearly resolve the complete length of the nanopillars, as their point of origin from the fiber surface is not distinctly discernible.

From Supplementary Figure 2 a-d, the direction of the nanostructures prove that the left side of these images was the face of the PDA dyed wool fabric that was plasma treated to create nanopillars. While the nanopillar structures are shown on one side of the fibers, fibers underneath the affected fibers are mostly untreated, showing that the ultrablack effect is mostly occurring from the very surface fibers of the fabric.

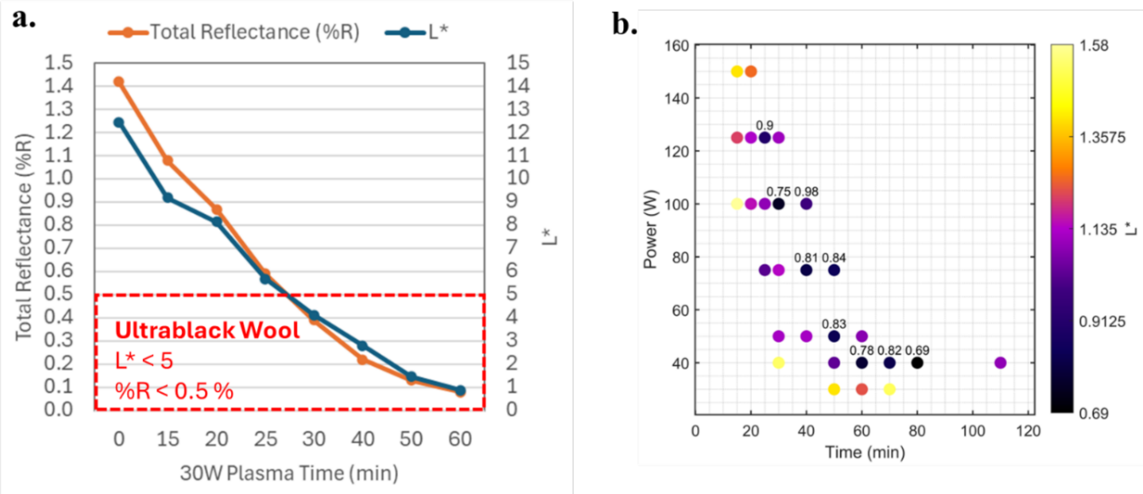

Supplementary Figure 3. a. Trendline of L* value and total reflectance of PDAMW. For PDAMW, it was identified that L* less than 5 would be categorized as ultrablack as the total reflectance (%R) of the samples was measured to be less than 0.5 %. **b.** Plasma treatment conditions for creating ultrablack wool from PDAMW. Labeled datapoints represent average L* values below 1.

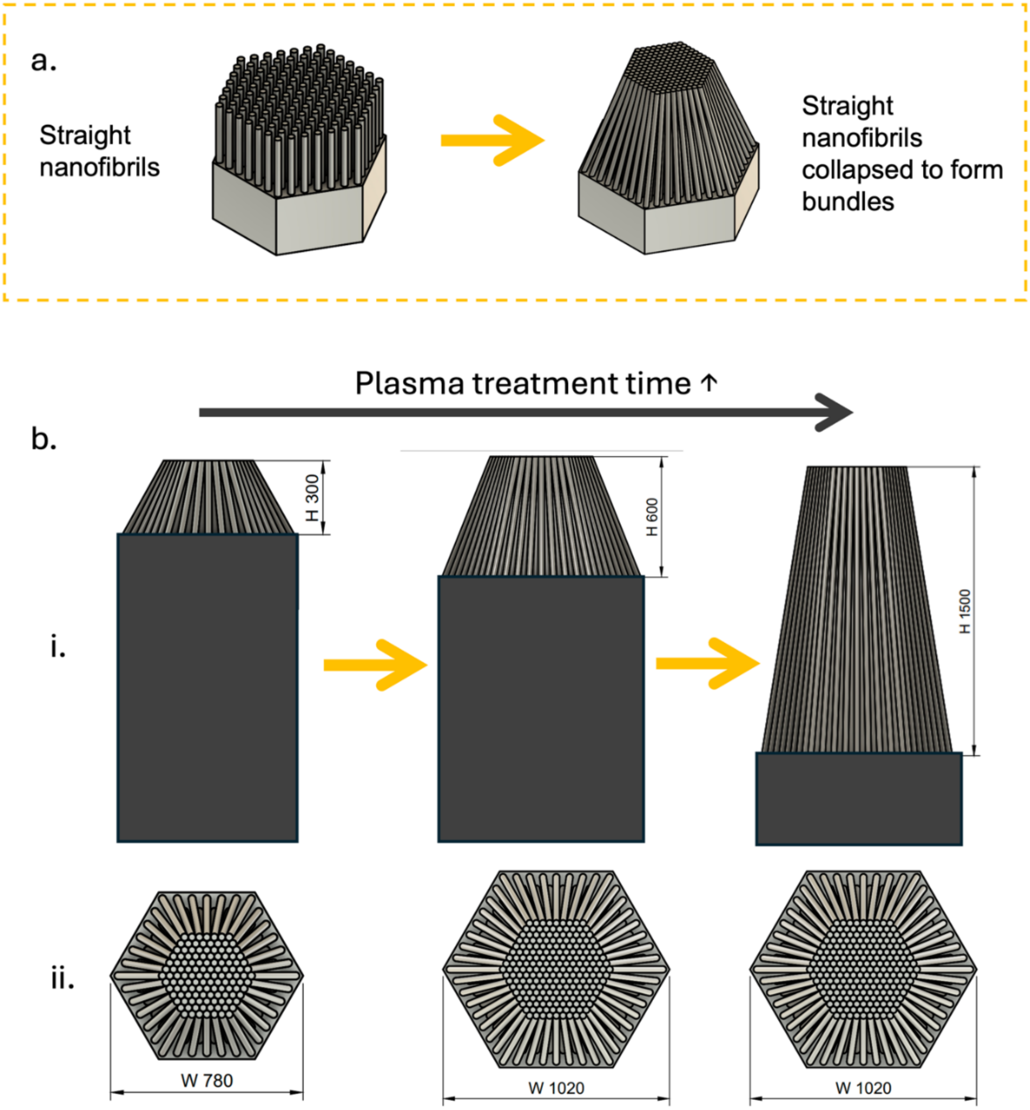

Supplementary Figure 4. a. Schematic drawing illustrating the bundles of nanofibrils observed on UBW being formed by collapsed straight nanofibrils. **b**. Schematic showing the progression of the structure as the plasma time is increased. i. side view of an individual bundle (xz plane) and ii. top view of a bundle (xy plane). We note that the height (*H*) as well as the width (*W*) of the bundles increase as the etching time increases.

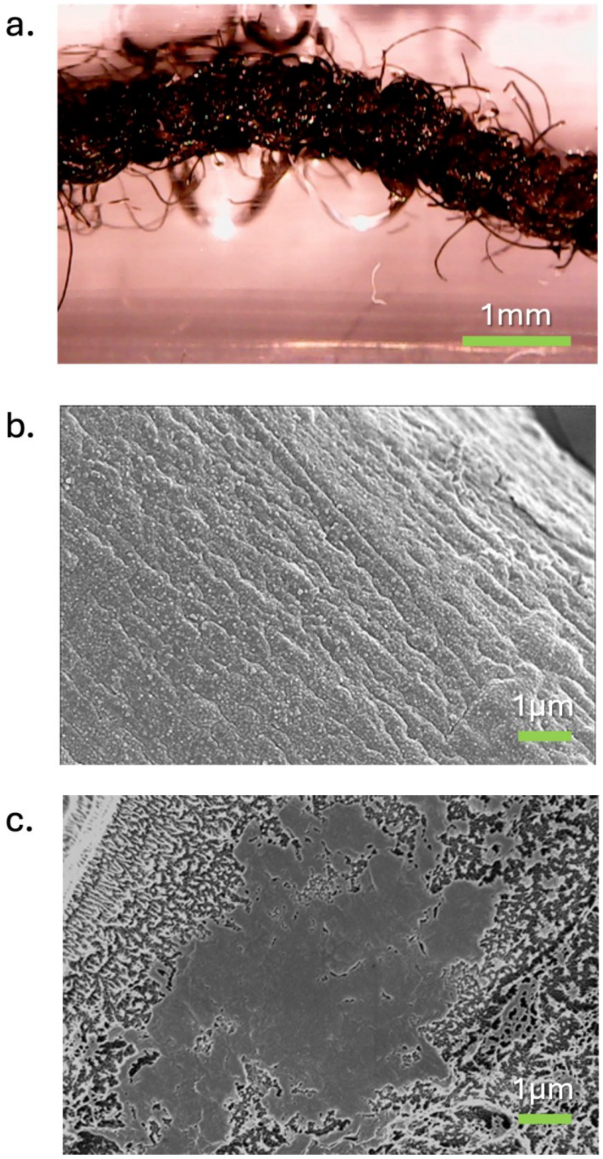

Supplementary Figure 5. a. Magnified image of the cross-section of the PDAMW fabric. **b.** PDAMW with surface coating of PDA nanoparticles and **c.** PDAMW etched for 5 minutes – the PDA nanoparticles on the surface have been completely removed and the surface has started to etch.

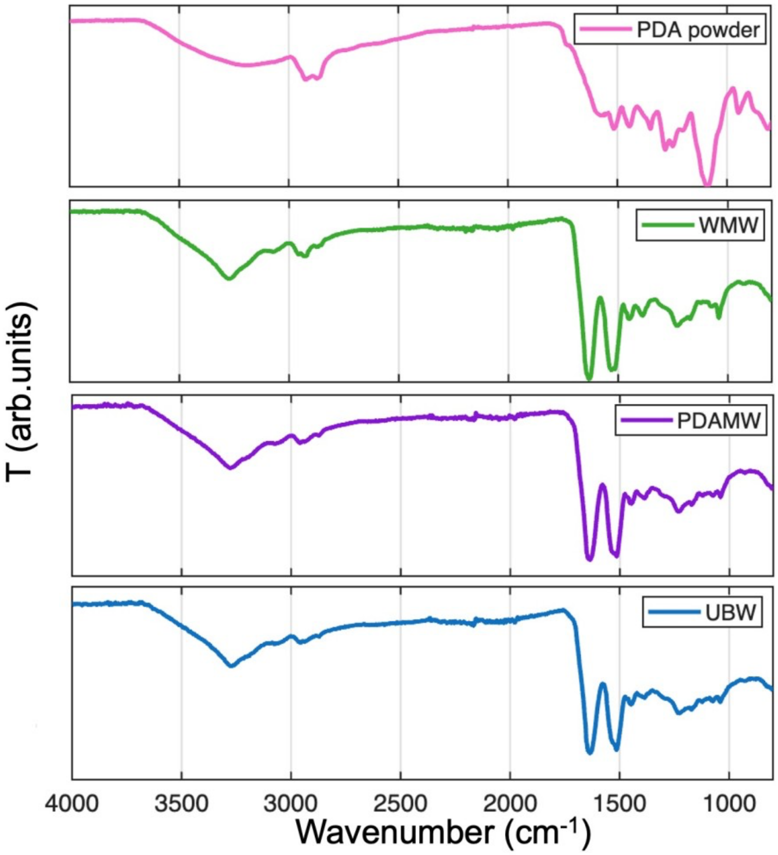

Supplementary Figure 6. The FTIR spectra of merino wool, polydopamine dyed merino wool (PDAMW), ultrablack wool (UBW) and polydopamine (PDA) powder. The spectra appear nearly identical in shape; however, detailed comparison reveals: i. peak position shifts, ii. the emergence of shoulder peaks and iii changes in peak intensities. The peak position shifts observed in amide I, II and III regions suggest that there are interactions between the polymeric structures of wool and PDA. The appearance of the shoulder peak at ~1713 cm^{-1} corresponding to the C=O in quinone confirms presence of PDA in the PDAMW and UBW. Additional characteristic vibrations of PDA include C=C (~1596 cm^{-1}) and C=N (~1510 cm^{-1}) stretching vibrations of the indole ring; however, these signals are overlapped by the broad amide I band of wool. Instead by normalizing the peak intensities at ~1530 and ~1515 cm^{-1} (normalized using the peptide backbone C=O stretching peak ~ 1634 cm^{-1}) for both merino wool and PDAMW, an increase in intensity is observed in the PDAMW peaks, suggesting the contribution from the indole ring of PDA. This confirms the presence of PDA in the PDAMW and UBW. The spectral peaks[1-4] and shifts are tabulated in Supplementary **Table 2**.

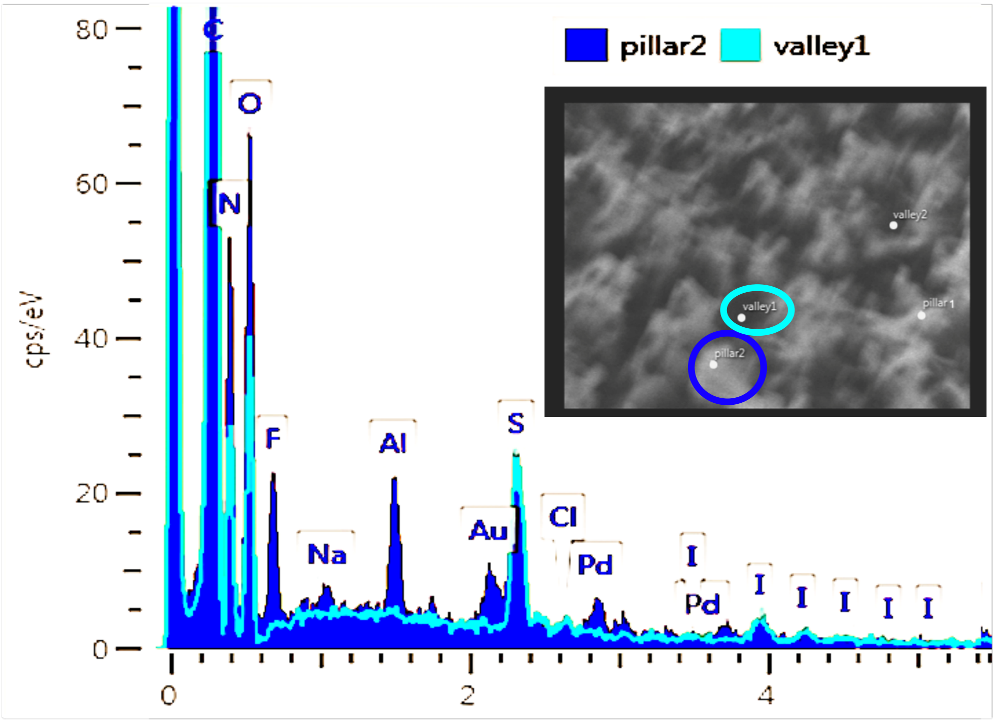

Supplementary Figure 7. SEM EDX spectra of the fiber surface to confirm metal co-deposition. Spot measurements were taken (for better accuracy) at pillar tops and valleys as shown in the SEM image (inset). The term co-deposited is used since deposition and etching both take place during the plasma process. The time span or lag (if any) between the two processes is unknown.

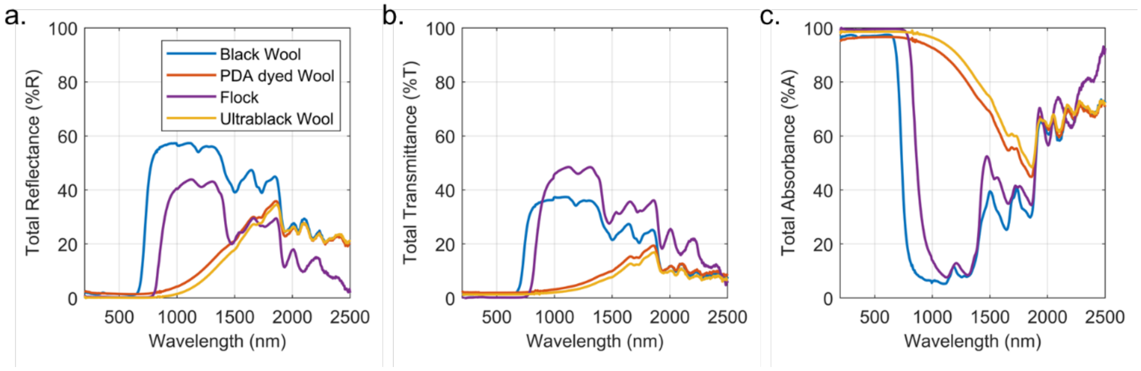

Supplementary Figure 8. a. Total reflectance, **b.** total transmittance, and **c.** total absorbance of black wool, PDAMW, flock, and UBW in full solar spectrum (λ = 200-2500 nm).

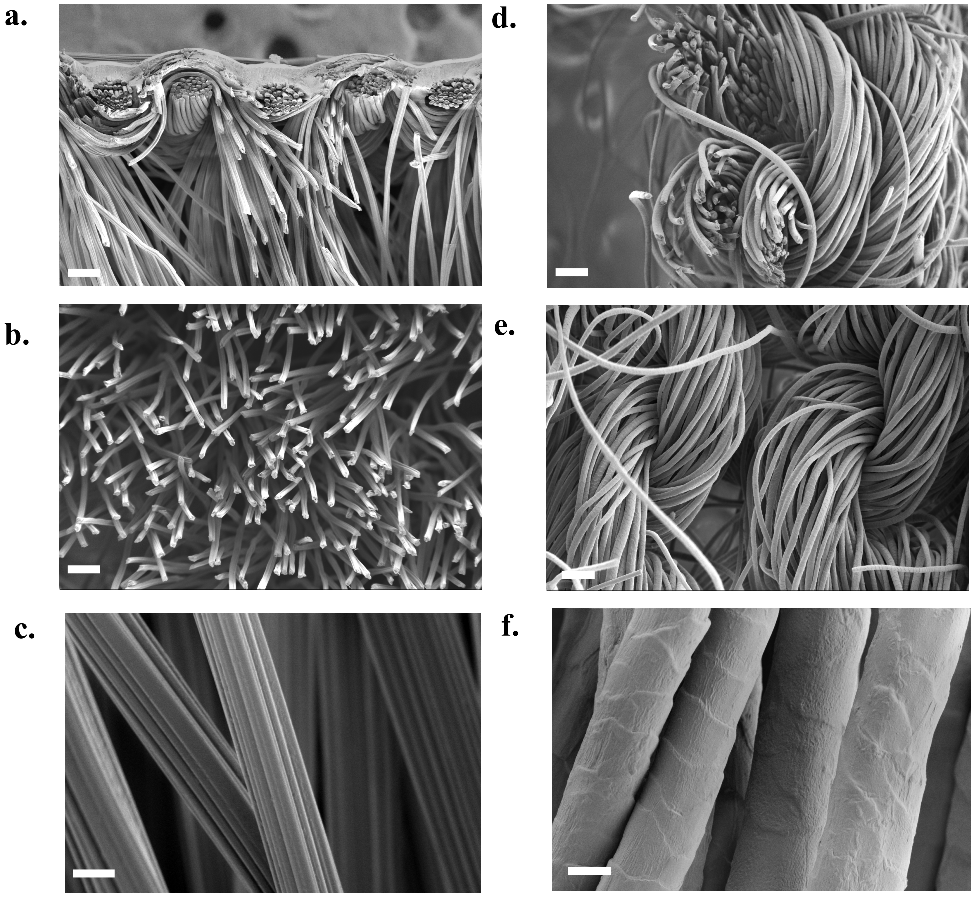

Supplementary Figure 9. SEM images of commercially available flock based ultra-black fabric (**a-c**) and UBW from this work (**d-f**). The images show the cross-section (**a, d**) of the fabrics, top view (**b, e**) of the fabrics (scale bar = 100 micron), and the surface of the fibers (**c, f**) (scale bar = 10 micron).

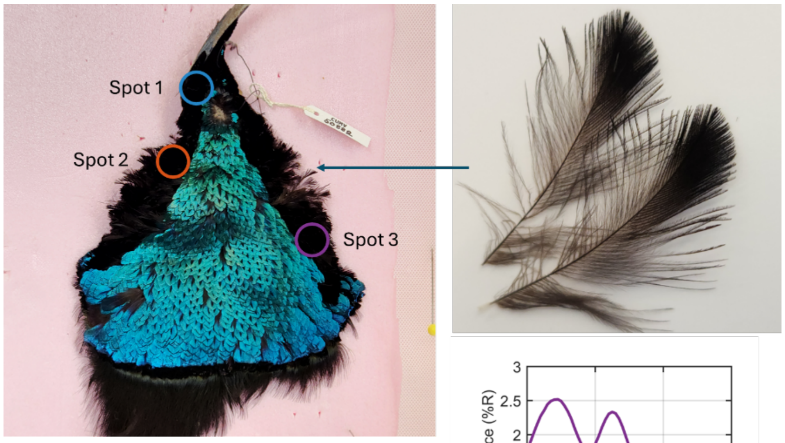

	%R (400-700nm)	%R (550nm)
Spot 1	0.61	0.52
Spot 2	0.78	0.95
Spot 3	1.51	1.83
Average	0.97	1.1

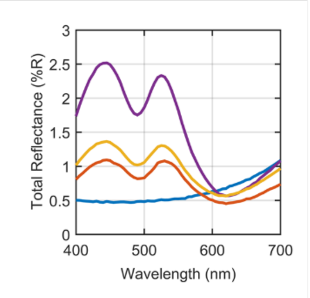

Supplementary Figure 10. a. Total reflectance (%R) measured on three corresponding (circled) spots of bird plumage. **b.** Summary of average %R (λ = 400-700 nm) and %R at 550 nm. **c.** %R of the three spots and the average of them, revealing the variation of orientation and their darkness depending on the position on the body. Plumage of a bird-of-paradise, *Ptiloris Magnificus* (Magnificent Riflebird), loaned from Cornell University Museum of Vertebrates (CUMV).

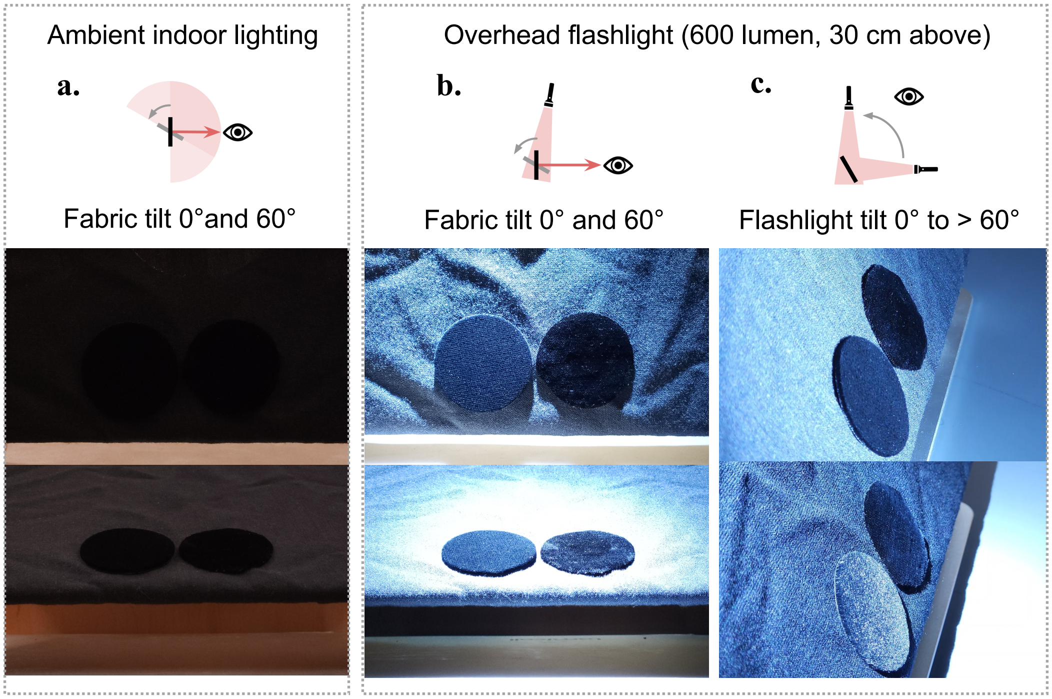

Supplementary Figure 11. Flock (left) and UBW (right) under various angles of incidence**. a.** Tilting the sample under ambient indoor lighting. **b.** Tilting the sample under flashlight. **c.** Tilting the flashlight to high angle of incidence when viewed from the side (refer Supplementary Video).

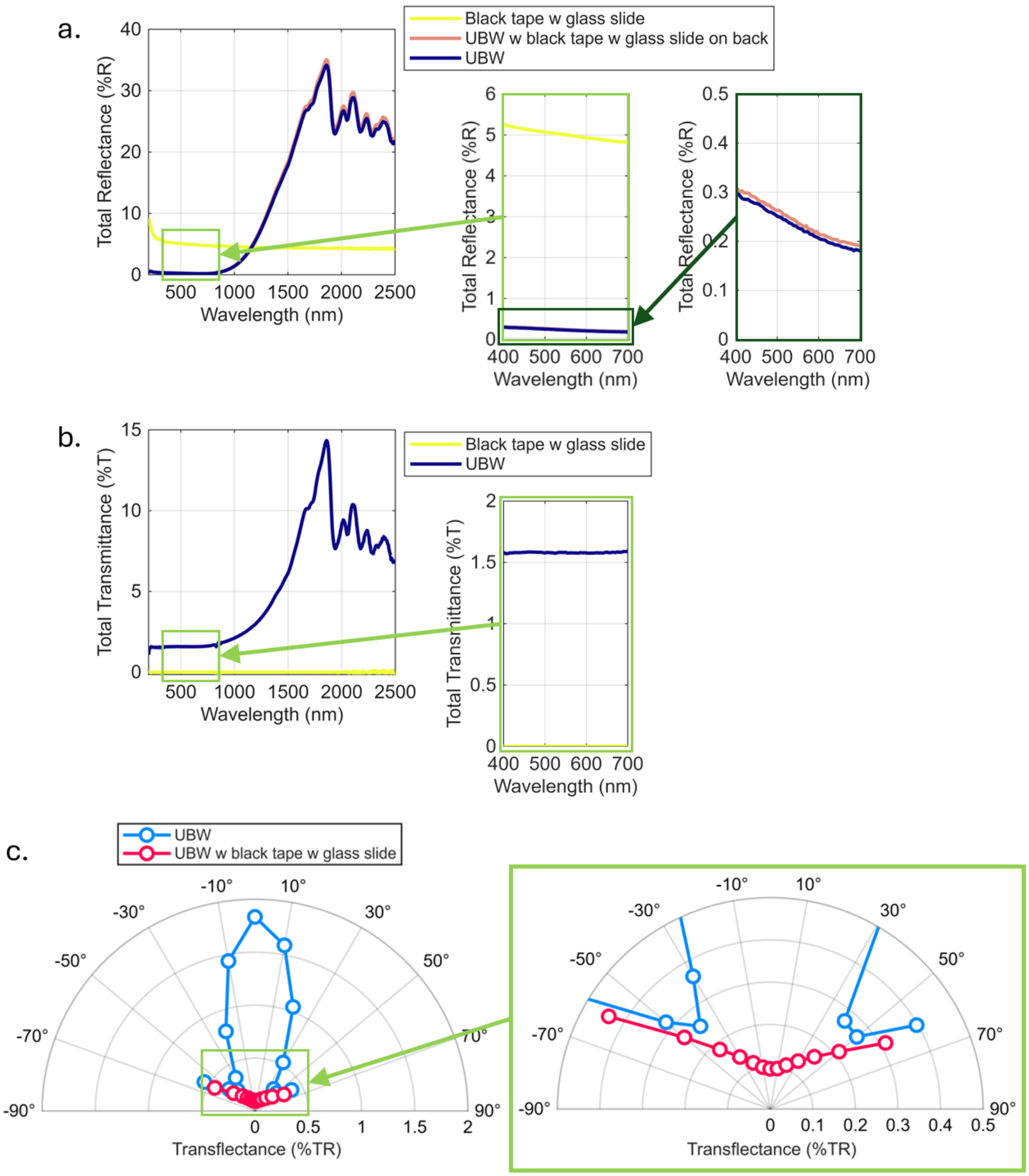

Supplementary Figure 12. a. Reflectance (%R) of UBW remains nearly identical, with or without the black backing material. **b.** Transmittance (%T) data shows that the black backing material can eliminate light transmission completely. **c.** With additional zero-transmittance backing material, UBW shows extremely low transflectance (%TR) at all probed angles.

Note: Slight variations in the metrics are expected, as the UBW sample used for these measurements above is different from the one presented in Figure 3.

a. b.

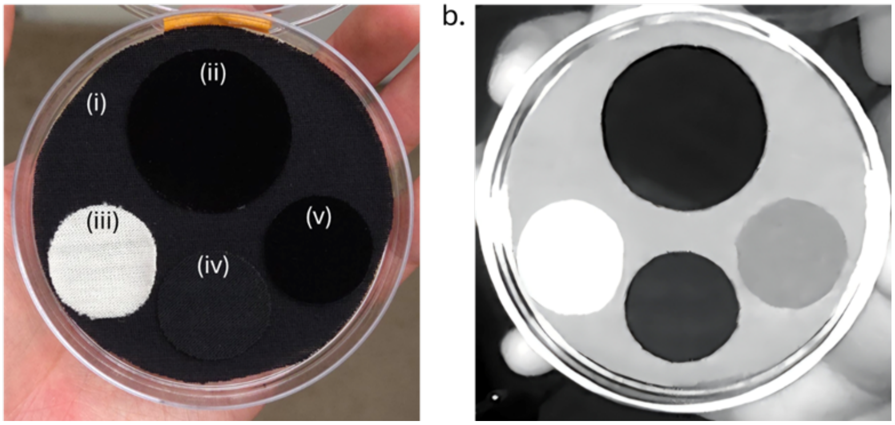

Supplementary Figure 13. a Photograph of (i) black wool, (ii) UBW, (iii) white wool, (iv) PDA dyed wool, and (v) flock fabric. **b** Near-IR reflectance imaging with night-vision camera.

Photographic images agree with spectrometric data in Supplementary Fig. 8 where UBW shows the darkest appearance from both regular camera and night-vision camera images compared to regular black wool, PDA dyed wool, and the commercially available ultrablack flock fabric.

When it comes to comparing UBW against NIR absorbing fabrics, we have purchased and analyzed the NIR absorbing fabric being sold by the same retailer that sold the flock fabric and included the result in Supplementary Fig. 14 to 16.

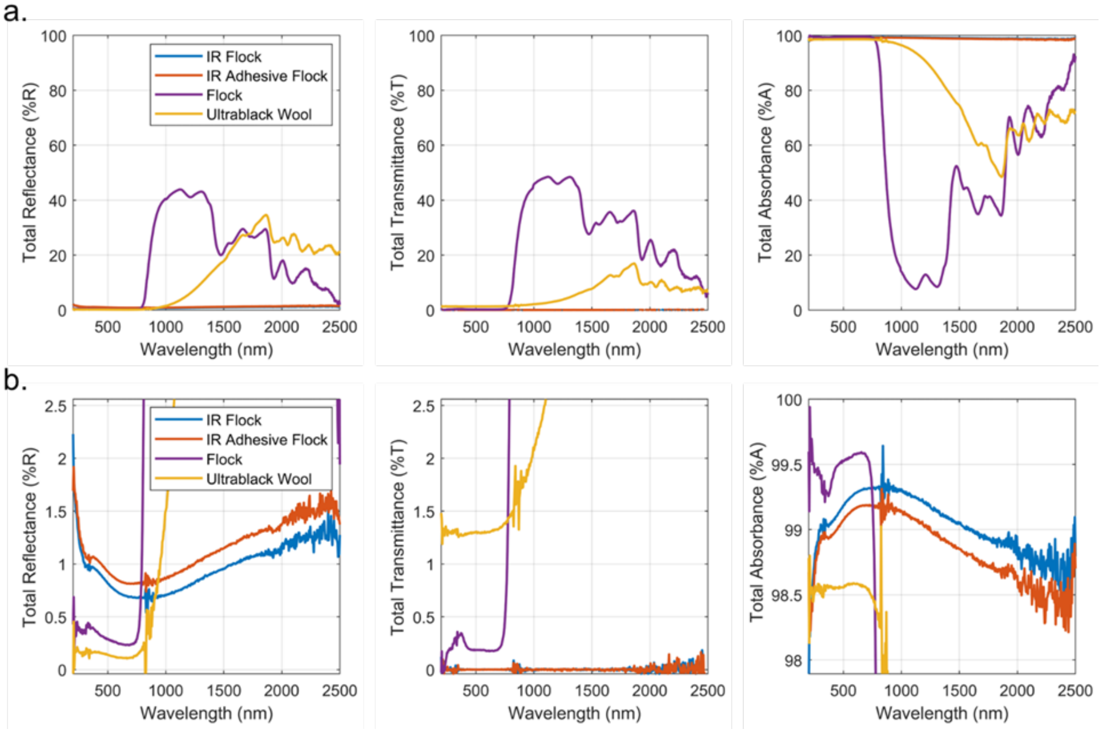

Supplementary Figure 14. a Total reflectance, total transmittance, and total absorbance of commercially available flock fabrics and UBW. **b** Zoomed-in plots.

All "flock" fabrics were purchased from Musou Black USA (https://musoublackusa.com/). IR Flock Sheet, IR Flock Sheet with adhesive, and Musou Black Fabric Kiwami (with back coat) are labeled as IR Flock, IR Adhesive Flock, and Flock, respectively, in the above figure.
The differences highlighted between the purchased (commercially available) and developed materials are presented purely as observational data to underscore the specific advantages and key findings of the present study. This analysis does not reflect on the overall quality or utility of the commercial products, which may perform optimally within their specified applications.

IR Flocks show low reflectance throughout the solar spectrum. The total reflectance within the visible spectrum, however, averages around 0.8~0.9%. Material with such reflectance would not be considered as "ultrablack". In comparison, the total reflectance of UBW within the visible spectrum is 0.13%.

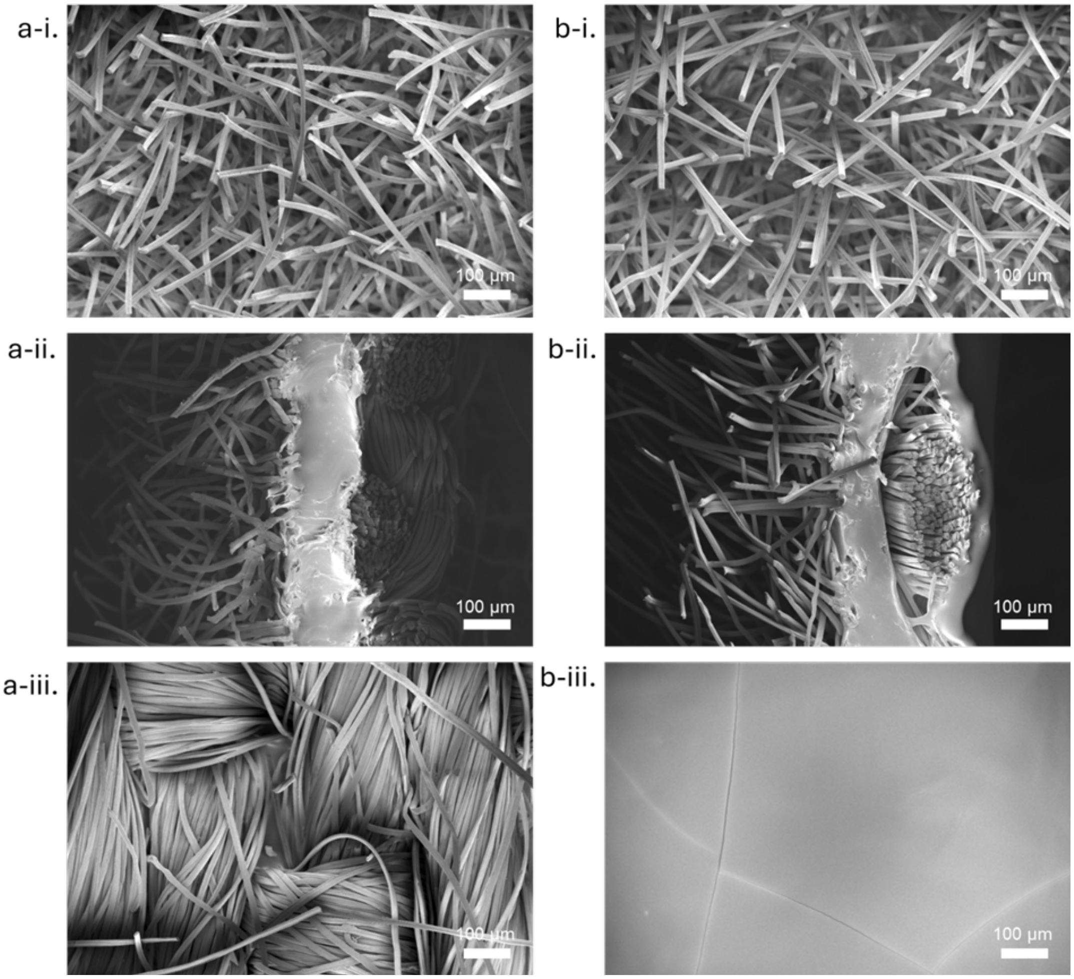

Supplementary Figure 15. SEM images of commercially available IR absorbing flock fabrics. Top view (**a-i**), cross-sectional view (**a-ii**), and bottom view (**a-iii**) of IR Flock Sheet and top view (**b-i**), cross-sectional view (**b-ii**), and bottom view (**b-iii**) of IR Flock Sheet with adhesive. Purchased from Musou Black USA (https://musoublackusa.com/).

SEM images confirm that all three flock fabrics comprise an underlying (middle or back) composite polymer layer, which contributes to their durability and/or the absorption of visible and/or near-infrared light. According to their website, the polymer mixture contains carbon substances.

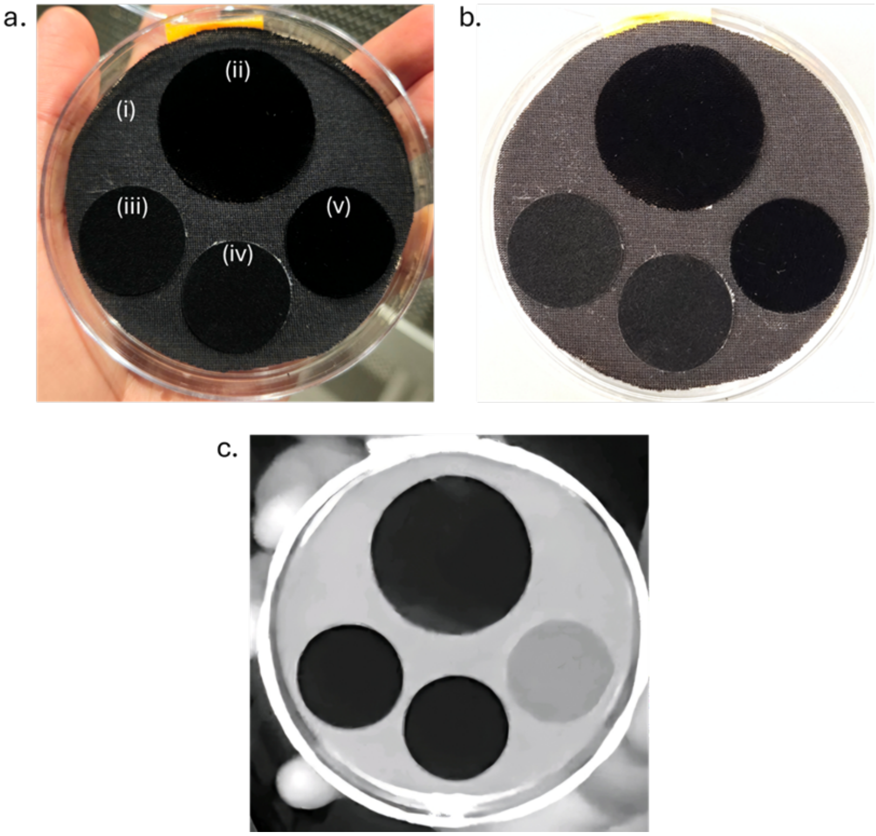

Supplementary Figure 16. a Photograph of (i) black wool, (ii) UBW, (iii) IR Flock, (iv) IR Flock with adhesive, and (v) flock fabric. **b** Photograph with increased exposure. **c** Near-IR reflectance imaging with night-vision camera.

Photographic images agree with spectrometric data in Supplementary Fig. S14, UBW showing the darkest appearance from regular camera images. As shown from spectrometric data, IR Flock exhibits high near-IR absorbance, appearing darker than UBW under night-vision image.

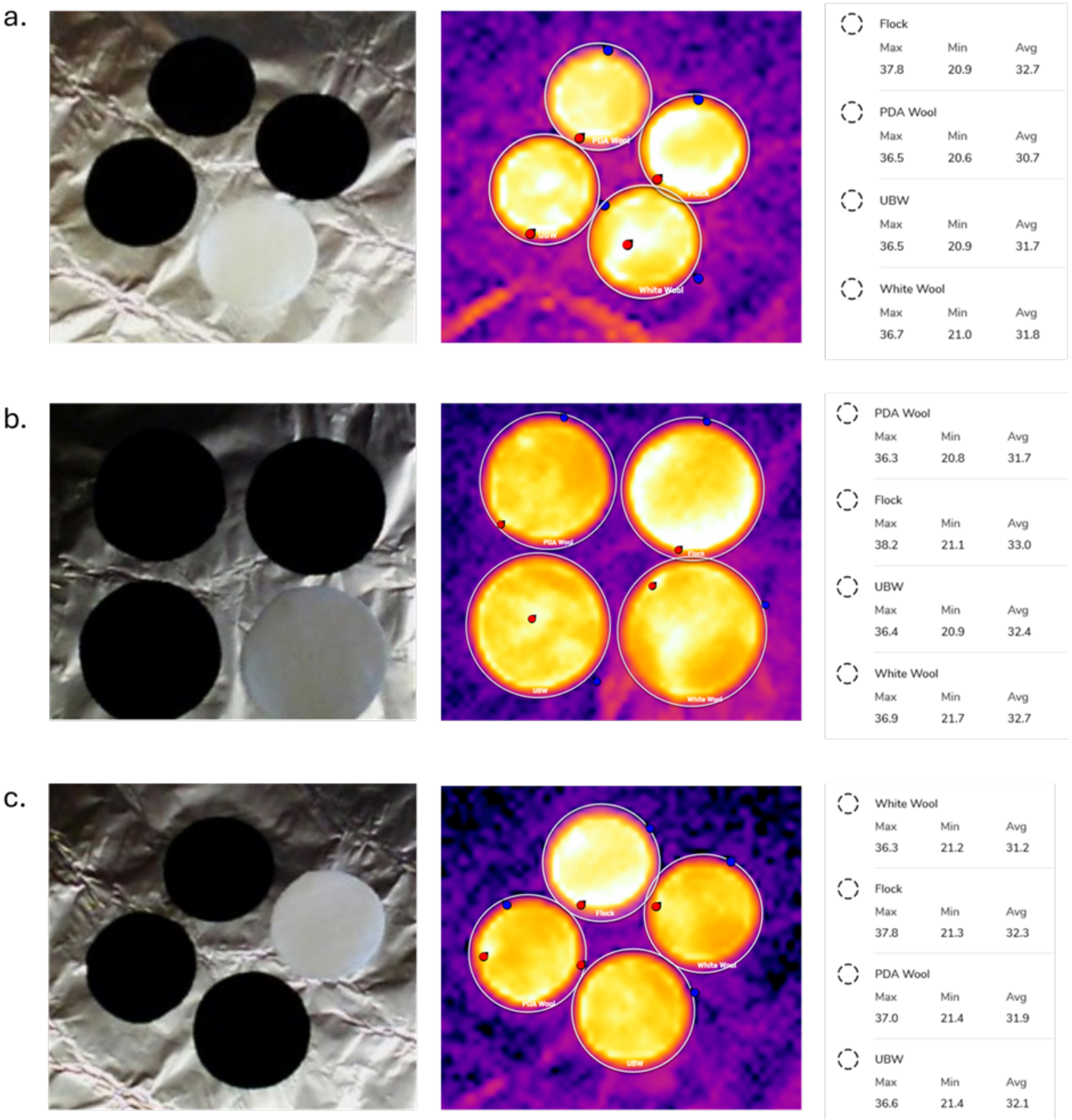

Supplementary Figure 17. Thermal imaging of white wool, PDA dyed wool, flock, and UBW. On hotplate heated to ~50°C, sample fabrics were placed with three different orientations/positions **a**, **b**, and **c**.

Thermal imaging indicates that neither PDA dyeing nor nanofibril fabrication for ultra-black wool (UBW) creation impacts the long-wavelength infrared (7-14 µm) emission. Flock fabric exhibited the highest maximum and average temperatures, likely attributable to its high gram per square meter (GSM) and the presence of a polymer coating layer.

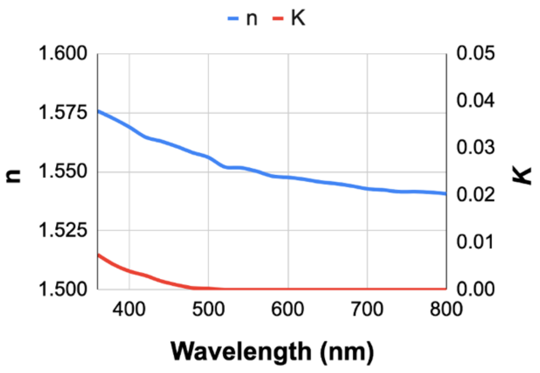

Supplementary Figure 18. Optical constants of keratin used for the FDTD simulations. n refers to the real part and *K* refers to the imaginary part of the complex refractive index.[5]

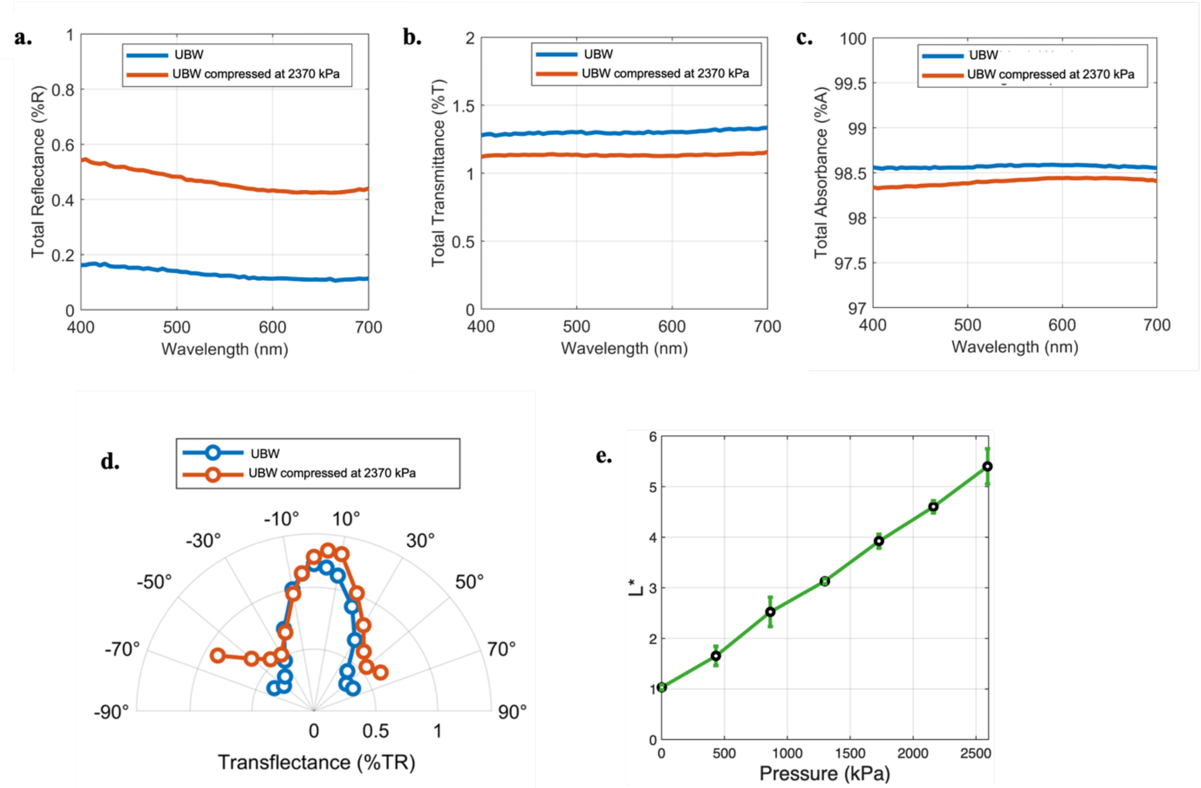

Supplementary Figure 19. a. Total reflectance (%R), **b**. total transmittance (%T), **c.** total absorbance (%A) within the visible spectrum (λ = 400-700 nm) and **d**. angle variable transflectance of UBW and UBW compressed at 2370 kPa, **e**. Change in average L* value as UBW is gradually compressed (standard deviations for each data point is marked).

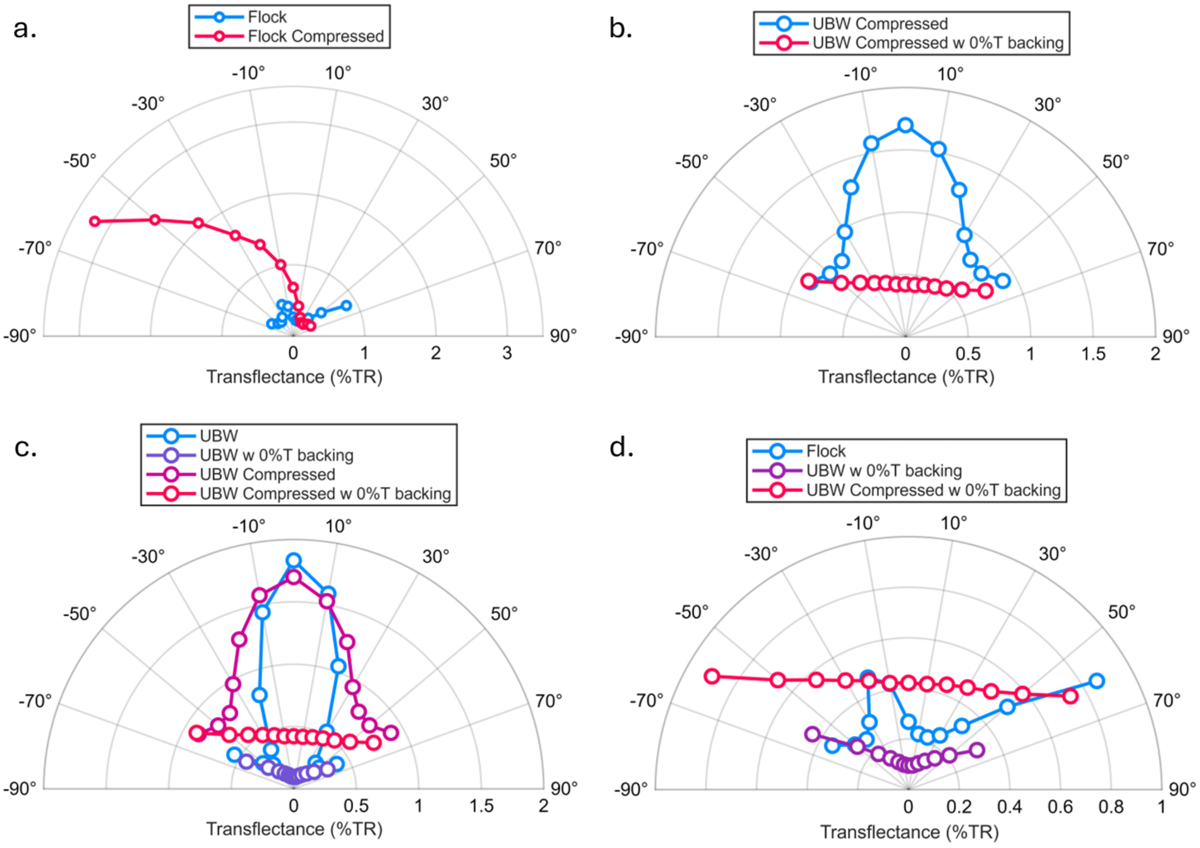

Supplementary Figure 20. Transflectance (%TR) comparison of compressed fabric. **a.** Flock fabric vs. compressed flock. **b.** Compressed UBW vs. compressed UBW with additional zero-transmittance backing material. **c.** Compressed UBW vs. uncompressed UBW (from Supplementary Fig. 12). **d.** Flock vs. UBW with backing material vs. Compressed UBW with backing material.

Supplementary Tables

Supplementary Table 1. Dimensional changes observed in the micro/nanostructures at plasma etching times 30 min, 50 min, 80 min and 110 min. The SEM images show that the pillars are made of multiple nanofibrils (diameter d 30 ± 9 nm)

	**30 minutes**	**50 minutes**	**80 minutes**	**110 minutes**
Height of nanofibrils H_{max} at the edge of fibers (nm)	1505 ± 184	1589 ± 263	3072 ± 373	–
Width of bundles W (nm)	456 ± 72	542 ± 96	796 ± 118	1141 ± 222
Gap between bundles (nm)	662 ± 116	596 ± 182	1406 ± 399	1597 ± 454
Diameter of pores D (nm)	55 ± 14	61 ± 9	69 ± 16	–

Notes:
*As observed in the SEM images of the fiber cross-sections (Supplementary Fig 2), the H at the top of the fiber cross section is shorter than at the boundary where the etching ends on the fibers' cross-sectional contour. The height H_{max} refers to the height of nanopillars/bundles at this edge.

Supplementary Table 2. Peak positions and shifts observed in the FTIR spectra for PDA powder, undyed wool, PDAMW and UBW fabrics.[1-4]

Functional group	Peak position (cm^{-1})				Peak position mentioned in literature (cm^{-1}) [1,2,3,4]
	PDA powder	Undyed wool	PDAMW	UBW	
N-H and O-H stretching	3664-2974	3654-3091	3651-3086	3660-3086	3200-3600
C-H stretching	2918, 2860	2953, 2918, 2867	2948, 2918, 2867	2952, 2918, 2867	2962, 2925, 2852
-C=O stretching in quinone or quinoid intermediary	1718	-	1713	1713	1720
-C=O stretching	-	1634	1639	1635	1600-1700 (amide I)
-C-N stretching and N-H bending	-	1515, 1532	1515, 1539	1515, 1534	1500-1600 (amide II)
C=C and C=N stretching of indole ring	1558, 1512	-	-	-	1596, 1510
C-N stretch and N-H bending	-	1223, 1069	1225,1070	1227,1071	1235, 1060 (amide III)
S=O stretching	-	1041	1040	1042	1030-1040

Supplementary Table 3: Chemical species identified by deconvoluting the high resolution XPS spectra for C 1*s*, N 1*s*, O 1*s* and S 2*p*. Peak assignment and analysis is done based on literature[6-9] and publicly available databases from Thermo Fisher Scientific and XPSfitting.com.

The WMW confirm the presence of abundant primary and secondary amino groups and oxygen species which ensure dyeability.[1] These primary amine groups and thiol groups present in wool can form covalent bonds with the residual catechol groups through mild Micheal additions or Schiff-base reactions.[10] The $\pi \rightarrow \pi^*$ shakeup satellite (~290.9 keV) of the C 1*s* peak is a common energy loss feature for aromatic carbon species and an indicator of the presence of PDA in both PDAMW and UBW. The aromatic $=NH_2$ in PDAMW and UBW suggests the presence of PDA. However, it should be noted that XPS is a surface technique (depth ~ 5nm), therefore, the sample PDAMW captures the wool surface as well as the PDA nanoparticles present on the wool surface, referring back to the Table 1 in the main manuscript, the N/C ratio of PDAMW is 0.125, which strongly hints at the presence of PDA on the surface. The S-S disulfide bonds (~163.94 keV) from sulfur containing moieties such as amino acid cysteine in UBW reconfirms the exposure of the protein below the lipid layer. The oxidized species such as O-C=O (~289.0 keV), NO_2^{-1} (~403.9 keV) and SO_4^{2-} (~171 keV) in UBW compared to WMW and PDAMW suggests plasma induced surface level oxidation. Especially, the S 2*p* photoelectron emission peak suggests the existence of multiple oxidation states from zero to +6.

Photoelectron peak	Chemical species	Binding energy (keV)	Atomic concentration (%)		
			WMW	PDAMW	UBW
C 1*s*	C-C,C-H,C-S	~284.8	45.69	28.98	24.86
	C-O,C-N	~286.4	41.19	44.16	43.07
	C=O	~288.5	13.11	25.07	21.65
	O-C=O	~289.0	-	-	7.86
	$\pi \rightarrow \pi^*$	~290.9	-	1.79	2.56
N 1*s*	aromatic $=NH_2$	~398.6	-	5.72	1.31
	R-NH-R	~399.9	93.43	75.78	72.19
	$R\text{-}NH_2$	~401.9	6.57	18.50	20.83
	NO_2^{-1}	~403.9	-	-	5.68
O 1*s*	C-O	~531.4	33.24	44.81	16.87
	C=O	~532.6	66.76	55.19	67.62
	adsorbed H_2O	~534.6	-	5.76	15.50
S 2*p*	S-S,S-C,S-H	~163.94	-	-	12.63
	SO_2/SO_3^{2-}	~168.31	100	100	66.89
	SO_4^{2-}	~171	-	-	20.49

Supplementary Table 4. The samples were listed in the order of increasing L* value starting with ultrablack wool (UBW), flock, black merino wool (BMW), black polyester blend (BPETB), PDA dyed merino wool (PDAMW), dark grey polyester (DGPET), PDA dyed Eri silk (PDAES),[11] and PDA dyed silk (PDAS).[12] The gradients of black in the Hex column show increased brightness and hue of colors when compared to UBW. The PDA dyed wool (PDAW)[1](last on the list) did not state the a* and b* value to generate the corresponding Hex code.

	Hex	L*	a*	b*
UBW	#020202	0.55	0.02	0.05
Flock	#040404	1.06	0.01	-0.11
BMW	#202022	12.39	0.53	-1.48
BPETB	#222122	12.87	0.75	-0.59
PDAMW	#222425	13.95	-0.09	-1.28
DGPET	#2C2D30	18.47	0.39	-2.37
PDAES	#352C24	18.6	2.92	6.66
PDAS	#3E3C3F	25.59	1.24	-1.33
PDAW	-	13.3	-	-

Supplementary Table 5. Average L* values of the UBW after applying compressive loads (kgf). Columns 2-3 provides the load in Pascals and psi units. The compression is applied using a 1/8'' steel disk of diameter 3.8 mm.

Load (kgf)	Pressure (kPa)	Pressure (psi)	L*
0	0	0	1.03 ± 0.05
50	432	62.68	1.65 ± 0.19
100	864	125.36	2.52 ± 0.29
150	1297	188.05	3.13 ± 0.05
200	1729	250.73	3.92 ± 0.14
250	2161	313.41	4.6 ± 0.12
300	2593	376.09	5.4 ± 0.35

Supplementary Table 6. Material and energy cost breakdown for manufacturing 1 m^2 of UBW.

	**Material quantity**	**Unit cost/$**	**Material cost/$**
Dopamine[1*]	16 g	3.947 per g	63.158
Sodium periodate	8 g	0.705 per g	5.642
water	8 L	0.00147 per L[2*]	0.0118
merino wool fabric[3*]	200 g	33.859 per m	33.858
		**Total**	**102.670**

	**Energy consumption[4*]**	**Unit cost/$**	**Energy cost/$**
Dyeing (heating)	0.186 kWh		0.0155
Drying fabric	0.667 kWh[5*]	0.0832 per kWh[6*]	0.0555
plasma etching	0.053 kWh		0.0044
		**Total**	**0.0754**
		**Total cost**	**102.745**

Notes:

[1*] The price is for purchasing 25 bottles (100 g each) of the chemical for lab scale use. This can further reduce for industry grade bulk purchases.

[2*] The unit price of water is per data available at www.epa.gov/watersense/data-and-information-used-watersense.

[3*] The price is for purchasing a roll of fabric and can further reduce for bulk purchases.

[4*] For calculating the energy consumption, we assume 100% efficiency.

[5*] Power of a large lab-scale dryer (2 kW) is used for the calculation.

[6*] The unit price of electricity is per data available at www. statista.com/statistics/190680/us-industrial-consumer-price-estimates-for-retail-electricity-since-1970/.

*For comparison we direct the readers to refer to the price of VACNTs grown on various substrates available at https://www.nano-lab.com/arrayindex.html. The price can be ≥ $450 for 1.25 mm x 1.25 mm VACNT grown substrate.

Supplementary Table 7. **a.** General comparison of UBW with commercially known darkest fabric (flock fabric) and VACNTs **b.** Comparison of textile properties of UBW and the flock fabric

**a.**	**UBW (our work)**	**Flock fabric**	**VACNT**
Process	Fabric dyeing followed by air plasma etching (dry technique)	Flock deposition via electrostatic interactions between fibers and fabric surface (dry technique)	Chemical vapor deposition (CVD) and other advanced techniques[13]
Material	Merino wool fabric	Rayon pile on rayon base fabric with/without binder	CNT
Material bioavailability	Yes. Sourced from sheep fiber	Yes. Regenerated from cellulose	No
Material availability	High	High	Low
Cost of production	Low	Low	High
Scalability	High	High	Low
Accessibility	High	High	Low. Require IP clearance
Toxicity	No	Not disclosed	Toxic metallic catalysts are known to be used for production of VACNTs during CVD, dimension and chemical residues can result in toxicity[14]

**b.**	**UBW (our work)**	**Flock fabric**
Flexibility	High	Low (with binder)
Breathability	High	Low
Density (gsm)	Low	High
R% of the textile (in Visible region)	0.13	0.274
Angle-independent reflectance	Yes	No
Dyeing process	Polydopamine dye (biocompatible[15])	Not disclosed
Sustainability-related facts	Wool is natural and biodegradable. Ensuring responsible and cruelty-free sourcing is important.	Ensuring sustainable deforestation is important. Harsh chemicals are used for regenerating cellulose. Binder used is not biobased

Supplementary References

1. Huang, H.; Zhang, W.; Han, X.; Han, Z.; Song, D.; Li, W.; Li, Z.; Wang, Y.; Xu, W. Effect of Polydopamine Deposition on Wool Fibers on the Construction of Melanin. *J Appl Polym Sci* 2023, *140* (5). https://doi.org/10.1002/app.53396.

2. Zhao, P.; Xu, P.; Zhang, X.; Du, Y.; Lei, J.; Xu, S. Effect of Polydopamine-Modified Protease on Shrink-Resist Properties of Wool Fiber. *Journal of the Textile Institute* 2024, 1-8. https://doi.org/10.1080/00405000.2024.2422676.

3. Rehman, A.; Houshyar, S.; Mirabedini, A.; Cheng, D.; Cai, G.; Padhye, R.; Wang, X. Durable, Lightweight, Washable and Comfortable Cooling Textiles from Nanodiamond/Polydopamine/Wool Nanocomposites. *Macromol Mater Eng* 2022, *307* (5). https://doi.org/10.1002/mame.202100856.

4. Zangmeister, Rebecca A., Todd A. Morris, and Michael J. Tarlov. Characterization of polydopamine thin films deposited at short times by autoxidation of dopamine. *Langmuir 2013,* 29 (27), 8619-8628. https://doi.org/10.1021/la400587j

5. Wilts, B. D.; Michielsen, K.; De Raedt, H.; Stavenga, D. G. Sparkling Feather Reflections of a Bird-of-Paradise Explained by Finite-Difference Time-Domain Modeling. *Proc Natl Acad Sci USA* 2014, *111* (12), 4363–4368. https://doi.org/10.1073/pnas.1323611111.

6. Qie, Runtian, Saeed Zajforoushan Moghaddam, and Esben Thormann. Parameterization of the optical constants of polydopamine films for spectroscopic ellipsometry studies. *Physical Chemistry Chemical Physics* 2021 23 (9), 5516-5526.

7. Briggs, D. and Beamson, G. XPS studies of the oxygen 1*s* and 2*s* levels in a wide range of functional polymers. *Analytical chemistry* 1993, *65*(11), pp.1517-1523.

8. El-Zawahry, M.M., Ibrahim, N.A. and Eid, M.A. The impact of nitrogen plasma treatment upon the physical-chemical and dyeing properties of wool fabric. *Polymer-Plastics Technology and Engineering* 2006, *45*(10), 1123-1132.

9. Meade, S.J., Dyer, J.M., Caldwell, J.P. and Bryson, W.G. Covalent modification of the wool fiber surface: Removal of the outer lipid layer. *Textile Research Journal* 2008, *78*(11), 943-957.

10. Jiang, Yin, Yue Lan, Xianpeng Yin, Haowei Yang, Jiecheng Cui, Tao Zhu, and Guangtao Li. Polydopamine-based photonic crystal structures. *Journal of Materials Chemistry* 2013 *C* 1(38), 6136-6144.

11. Leamkaew, V.; Jitjankarn, P.; Chairat, M. Polydopamine-Dyed Eri Silk Yarn for the Improvement of Wash and Light Fastness Properties. *Journal of the Textile Institute* 2021, *112* (4), 553–560. https://doi.org/10.1080/00405000.2020.1771121.

12. Yan, B.; Zhou, Q.; Zhu, X.; Guo, J.; Mia, M. S.; Yan, X.; Chen, G.; Xing, T. A Superhydrophobic Bionic Coating on Silk Fabric with Flame Retardancy and UV Shielding Ability. *Appl Surf Sci* 2019, *483*, 929–939. https://doi.org/10.1016/j.apsusc.2019.04.045.

13. Hughes, K.J., Iyer, K.A., Bird, R.E., Ivanov, J., Banerjee, S., Georges, G. and Zhou, Q.A., 2024. Review of carbon nanotube research and development: materials and emerging applications. *ACS Applied Nano Materials*, *7*(16), pp.18695-18713.

14. Awasthi, S., Srivastava, A., Kumar, D., Pandey, S.K., Mubarak, N.M., Dehghani, M.H. and Ansari, K., 2024. An insight into the toxicological impacts of carbon nanotubes (CNTs) on human health: A review. *Environmental Advances*, *18*, p.100601.

15. Kohri, M. Progress in polydopamine-based melanin mimetic materials for structural color generation. *Science and Technology of Advanced Materials* 2020, *21*(1),833-848.
