# Supplementary material for: Ultrablack wool textiles inspired by hierarchical avian structure
Source: Nat Commun. 2025 Nov 26;16:10581. doi: 10.1038/s41467-025-65649-4 (PMC12657505; doi:10.1038/s41467-025-65649-4)
Supplement: Supplementary file 2 — Description of Additional Supplementary Files [file 41467_2025_65649_MOESM2_ESM.pdf]

**Description of Additional Supplementary Files**

File Name:

**Supplementary Movie 1**

Description:

**Visual comparison of light reflection on Ultrablack Wool and Flock fabric.** A flock fabric (left) and our ultrablack wool (UBW, right) are illuminated by a 600-lumen flashlight from dynamic angles at close range (~30 cm) in a darkroom. With the fixed viewing angle, the flock fabric shows strong reflections while the UBW remains uniformly dark.
